# Maturation processes in glass-ionomer dental cements

**DOI:** 10.1080/23337931.2018.1497492

**Published:** 2018-07-31

**Authors:** John W. Nicholson

**Affiliations:** a Dental Physical Sciences, Institute of Dentistry, Barts & The London School of Medicine and Dentistry, Queen Mary University of London, London, UK;; b Bluefield Centre for Biomaterials, London, UK

**Keywords:** Glass-ionomers, Maturation, Strength, Secondary setting reaction, Opacity, Bound water, Bonding

## Abstract

Glass-ionomer cements are used for a variety of tooth-repair functions in clinical dentistry. They are formed by reaction of a basic glass powder with a solution of polymeric water-soluble acid, usually polyacrylic acid. After the initial neutralization reaction, by which the cement hardens, various maturation reactions occur. Changes induced by these maturation reactions are identified as: increase in strength; reduction in plasticity; improvement in opacity; and increase in proportion of tightly bound water. In addition, in contact with the tooth, an ion-exchange interfacial layer is gradually formed. This is mechanically strong and chemically-resistant. These changes are described in the current paper, which reviews the extent to which they occur, and reports what is know about the chemistry that underlies them. Processes involving slow diffusion of various ions and of water through the set cement bring about these changes. They include a secondary setting reaction to form a phosphate-based phase, binding of water to co-ordination sites around metal cations and to a hydration sheath around the polymer molecules, and possibly reaction of water with glass particle surfaces to form silanol groups. Evidence from a wide range of literature sources is used to be build up a detailed picture of the chemistry of the maturation processes, and gaps in our understanding are highlighted. The article concludes that, given the importance of glass-ionomers in contemporary dentistry, it is important to know the extent to which such maturation processes occur in current cement formulations, and also to determine how rapidly they take place.

## Introduction

Glass-ionomer cements are acid-base materials that are formed by the reaction of weak polymeric acids with basic alumino-silicate glass powders [1–3]. Modern versions of these materials typically comprise powders that contain some of the polymeric acid in dried form, so that the acid solution is not too viscous while allowing the freshly mixed cement to contain the high amounts of acid necessary for the achievement of rapid setting and high strength. This type of formulation is characteristic of the so-called “high-viscosity” glass-ionomers, a term typically applied to materials with powder:liquid ratios of at least 3.6 to 1.

The polymers employed in glass-ionomer cements are either homopolymers of polyacrylic acid or the copolymer of acrylic and maleic acid (monomer ratio 2:1) [3]. Other monomers have been suggested for inclusion in polymers for cements [4], but none are used in commercial materials. The alternative polymer polyvinyl phosphonic acid is used in practical glass-ionomer cements [5], but as a rate-modifier in a blend with polyacrylic acid in a single commercial brand.

Glass-ionomer cements are versatile materials, whose uses in clinical dentistry include as full restorative materials, notably in children, liners and bases, fissure sealants and bonding agents for orthodontic brackets [3]. They are the material of choice for the Atraumatic Restorative Treatment (ART) technique [6, 7], in which application they have shown high durability and good clinical outcomes over several years [8].

## Changes on maturation

Glass-ionomer cements undergo a rapid initial hardening reaction, but continue to undergo changes for some time after this hardening is complete. These later processes are known jointly as *maturation*, and they are the subject of this review paper. In this paper, the key changes are identified, and what is known about their underlying chemistry is described.

The initial setting reaction is a neutralization between the aqueous polymeric acid solution and the glass powder [3]. This reaction forms calcium (or strontium) polyacrylate immediately, with aluminium polyacrylate forming slightly later [9]. The cement includes a substantial amount of unreacted glass particles, which act as reinforcing filler in the polymeric matrix. Setting takes place rapidly, typically in time periods of 2–6 minutes.

Once this initial setting is complete, several changes take place over the following days or even months. They include the following:

- Compressive strength typically increasing asymptotically to a stable value higher than the one found at 24 hours;
- Toughness declining and the cement becoming more brittle;
- Opacity declining and translucency increasing;
- The proportion of bound water increasing to a limiting value;
- Ion exchange bonding to the tooth surface developing with time.

These processes are understood to different extents. Also, in certain cases, much of the data comes from very early studies on these materials, and it is not always clear that modern glass-ionomers change to the same extent, or take so long to reach final values. This aspect is discussed in the later sections of this paper.

These changes are generally considered to be advantageous from the point of view of the clinical performance of glass-ionomers [1]. The increase in strength is clearly desirable, as is the improvement in opacity, with corresponding improvement in aesthetics. The binding of water per se is not of any particular value, but the resulting reduction in susceptibility to water loss clearly is, as it means the cement no longer needs protection with varnish or petroleum jelly. Once water-binding has occurred, cements will no longer lose water forming micro-cracks and developing an unsightly chalky appearance.

There have been attempts to accelerate these maturation processes, for example by the application of heat from a dental cure lamp or ultrasound from an ultrasonic device [10]. Both of these techniques have been shown to improve the early strength of glass-ionomer cements [10, 11]. However, no information has been published on how these techniques affect either opacity or water-binding.

## Secondary setting reaction

That glass-ionomer cements consist of more than simply ionically cross-linked polyacid molecules reinforced by unreacted glass particles was first proposed more than 25 years ago [12]. This arose from the finding that hard, insoluble cement materials could be made by reacting ionomer glasses with ethanoic (acetic) acid [12]. Later it was shown that similar cement materials could be made using lactic acid [13]. These materials were later called pseudo-cements, a helpful term because it distinguishes them from the clinically useful proper glass-ionomer cements made with polymers [14].

The formation of pseudo-cements is important because the relevant salts (e.g. calcium acetate) that must form initially are all readily soluble in water. Despite this, pseudo-cements that have been aged for at least 6 hours, and preferably 24 hours, do not dissolve in water. From this observation, and the fact that infrared spectroscopy was unable to detect any differences between pseudo-cements that were 1 hour old (and still soluble in water) and those that were 24 hours old, led to the suggestion that an inorganic network had formed, derived from the ion-depleted material left over from the reacted glass [12]. Such a network would contain no species capable of absorbing in the relevant region of the infrared spectrum, and thus show no differences in the infrared region as it formed.

The initial suggestion was that the additional network was some sort of silicate species. However, subsequent work on a range of ion-leachable glasses showed that insoluble pseudo-cements were formed only with phosphate-containing glasses [14]. Phosphate-free glasses were able to react with acetic acid, but the resulting cements never became insoluble in water. From this, it was concluded that the proposed inorganic network is, in fact, some sort of phosphate species [14].

The formation of this phosphate network has been advanced as the reason that glass-ionomers become less tough and more brittle as they age [12, 15]. This behaviour is in distinct contrast with that of zinc polycarboxylate cements [15]. They are closely related materials, but they can only consist of ionically cross-linked polyacid chains with unreacted filler embedded in them. There is no possible secondary reaction. As a result, zinc polycarboxylate cements remain relatively tough (plastic) for their whole existence, with no increase in brittleness once the neutralization setting reaction is complete [15].

A further contribution to the secondary setting reaction is the change in co-ordination number of aluminium [16]. Aluminium is known to be present in the glass predominantly in 4-co-ordination, though there are minor amounts in 5-co-ordination in some glasses. As setting proceeds, it changes its co-ordination state and becomes predominantly 6-co-ordinate [16]. This is readily understood in terms of the chemistry of aluminium, since the ability to co-ordinate with a variety of species is well known, and within the cement, it has the possibility of co-ordinating with carboxylate groups on the polymer, water molecules and fluoride ions. There is also evidence that, as the co-ordination state slowly changes over the first few weeks of a cement’s existence, so some sort of Al-O-P species forms as well [16]. This may be in addition to the main phosphate network, or it may be an integral part of its structure.

## Changes in strength

The standard technique for measuring the strength of conventional glass-ionomer cements is in compression [17]. However, other types of strength have been used, namely flexural [18], biaxial flexure [19], diametral tensile [20] and shear punch [21]. Most of the data on change in strength concerns compressive strength, and much of it is concerned with very early glass-ionomer formulations. There is relatively little published data on how the strength of modern glass-ionomers changes with maturation, though what there is suggests that these materials, too, become stronger with time over the initial few weeks after preparation.

**Figure 1.**
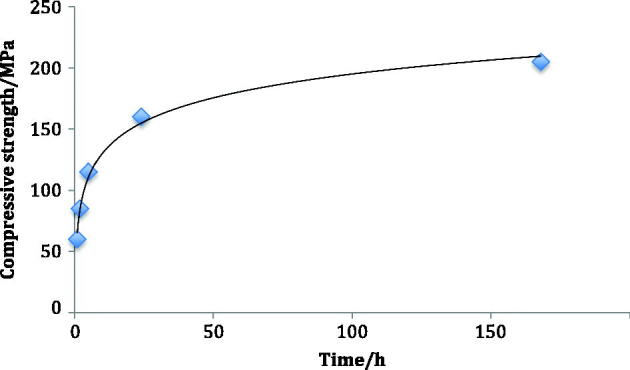
Increase in compressive strength (MPa) with time (hours) for early glass-ionomer cement formulation.

The observation that glass-ionomer cements become stronger in compression as they mature was first made by Crisp et al in 1976 [22]. Selected data from their study are shown in Figure 1. As can be seen, there is gradual increase in strength that approaches a limiting value, which is achieved at about 1 month.

Not all glass-ionomers behave in this way. Cements made from the acrylic-maleic acid copolymer reach a maximum compressive strength at around 3 weeks, then decline slightly to a limiting value [23]. This was attributed to hydrolytic instability [23]. However, it is difficult to see why this should be the case from the point of view of the chemistry of these materials. There is no obvious reason why ionic cross-links should be susceptible to hydrolysis when formed from acrylic/maleic acid copolymer, yet stable when formed from the homopolymer of acrylic acid. Experimental studies have confirmed that this is not caused by hydrolysis, because similar effects have been found when cements based in acrylic/maleic acid copolymer are stored in anhydrous conditions in vegetable oil (see Table 1). Under these conditions, cements reached maximum strength at 1 week, and declined significantly in strength by 4 months [24].

**Table 1.** Compressive strength of glass-ionomer (Chelon-Fil, ESPE, Germany), mixed at 1.3:1 powder:liquid ratio and stored in vegetable oil (Standard deviations in parentheses).

Storage time	Compressive strength/MPa
24 h	188.8 (15.2)
1 week	199.4 (12.0)
1 month	181.5 (15.3)
2 months	154.4 (16.2)
3 months	159.8 (12.3)
4 months	124.5 (20.3)

When, later, experimental cements formed from polyvinyl phosphonic acid were found to show similar changes in compressive strength (Table 2) [25], an alternative physical explanation was proposed. This is that both acrylic/maleic acid copolymer and polyvinyl phosphonic acid have higher cross-link density than polyacrylic acid, and this eventually creates cements that are more brittle (and less plastic) in character than those made from polyacrylic acid. Highly brittle materials tend to be more sensitive to surface flaws and other imperfections than tougher ones. Hence, it was suggested that the increasing brittleness of cements made from acrylic/maleic acid copolymer and also from polyvinyl phosphonic acid made them become slightly weaker with time [25].

**Table 2.** Compressive strength of experimental glass-ionomer cement based on polyvinyl phosphonic acid, mixed at 3:1 powder:liquid ratio (*n* = 6 per time period) and stored in water (Standard deviations in parentheses).

Storage time/days	Compressive strength/MPa
1	84 (15)
30	146 (25)
90	112 (9)

Although the strengths (compressive, diametral tensile and/or biaxial flexure) of modern and experimental glass-ionomer cements have been widely reported, there are few more recent studies of how these properties change with age. One exception was the report by Bresiani et al [26], which showed that, for three modern formulations, both compressive and diametral tensile strength improved with age up to 1 week. The change between 1 hour and 24 hours was large and statistically significant in all cases. By contrast, although each material showed a slight increase between 24 hours and 7 days, in no case was the change significant. This suggests that the extent of changes on maturation reported for the earliest formulations of glass-ionomer may not occur to the same extent in modern materials, though further work is need to confirm this point.

The increase in strength of most glass-ionomers is mirrored in the behaviour of pseudo-cements. Those made from acetic acid gradually increased in strength (Table 3), reaching about 100 MPa in compression after 3 months [12]. The materials were very fragile, and clearly brittle, despite the high compressive strength. This suggests that at least part of the increase in strength of conventional glass-ionomer cements is due to the gradual formation of this phosphate phase as part of the secondary setting process.

**Table 3.** Compressive strength of pseudo-cements made from 45% acetic acid with ionomer glass (powder:liquid ratio 4:1) (Standard deviations in parentheses).

Storage time	Compressive strength/MPa
1 day	11.9 (3.3)
1 week	24.8 (14.3)
1 month	72.0 (27.7)
3 months	104.4 (25.2)
6 months	104.5 (25.3)

Recently evidence has been found that these increases in strength correlate with decreases in apparent porosity with time, as determined using a combination of neutron beams and X-rays [27]. In the past, the existence of pores within glass-ionomer cements has been observed and attributed to air-entrainment during the mixing process [28, 29]. Certainly the size and distribution of these pores varies depending on whether the cements are mixed by hand, using a spatula to incorporate the glass powder into the liquid, or automatically, in a capsule, using a vibratory mixer the physically force the powder and liquid to combine [28].

Evidence has emerged recently that these “pores” may not, in fact, be air bubbles at all, but translucent gel structures capable of further slow further reaction [30]. Zinc phosphate cement has been shown to contain what look like air voids but which turn out to have measurable composition of zinc and phosphate ions. This apparent porosity develops by liquid segregation not by air entrapment during mixing, and there were fewer such structures in cements prepared with less liquid.

In principle, similar “air-voids” in glass-ionomers may also form by the same process of liquid segregation, and it is likely that such structures also contain gels, albeit of a different chemical composition from those in zinc phosphate cements. Such gel structures could mineralise gradually from the edge inwards as the relevant ions diffuse into them. In this way, they would shrink with time. The resulting reduction in volume of these structures could then improve the strength of the ageing cement, and thus be one of the mechanisms by which compressive strength increases in maturation. Further work is required to explore this possibility.

## Change in opacity

By comparison with composite resins, glass-ionomers show poor translucency and inferior aesthetics [31]. However, unlike all other dental cements, modern materials do have a degree of translucency, and this changes with time during the maturation phase, so that after 24 hours translucency is much improved [32]. Early glass-ionomer materials were relatively opaque due to the high fluoride content of the glass powder used, but this is one of the properties that have been improved in contemporary glass-ionomers.

The two properties of opacity and translucency are related, in that a material with high opacity has low translucency. Matching the appearance of the tooth requires restorative grade glass-ionomers to match, as far as possible, the translucency of the tooth. However, despite the improvements in modern materials, they are still typically more opaque (less translucent) than the natural tooth [32].

In principle it is possible to measure opacity in the laboratory. Opacity is not a material property, because it depends on other features of the measurement conditions, notably background colour [32]. It can be quantified in terms of the contrast ratio, C_r_ defined as:

$$\text{Cr}=R_{0}/R_{r}$$

where the R values are measures of reflected light when a specimen of material of defined thickness, in this case 1 mm, is placed on a background surface. R_0_ is the value when the specimen is on a black background and R_r_ is the value on a white background. For dental cements, the reflectivity of the white surface used is 0.7 (i.e. 70%) of that of a “pure” white surface, so these values are known as C_0.7_ values.

Using this approach, the translucency of the tooth enamel is around 0.55 (though it declines as the tooth darkens with age), and the desirable value for an aesthetic repair material is considered to be below this [31]. Early glass-ionomers were found to have values 0.60–0.85, an indication of their relative opacity [33]. These values became slightly lower with time, reaching a limiting value some time between 1 day and 1 week [33]. However, they were still above the ideal, and even modern glass-ionomers are not able to reach the desired degree of translucency.

In recent years, it has become unusual to measure this value. Instead, images of cement specimens placed on black-and-white zig-zag lines tend to be presented. This is not a quantitative method, but does give a much better idea of the material’s appearance than the numerical approach. There has been a general improvement in translucency in modern materials with the exception of Chemfil Rock (Dentsply, Germany). This brand is made from a novel zinc-containing glass of high opacity, and the resulting cement shows relatively poor appearance. However, mechanical properties are good [34], and the material continues to find clinical application, despite its relatively high opacity.

## Change in proportion of bound water

Water is an essential component of glass-ionomer cements, and has several functions in these materials [35]. It is the solvent for the dissolution of the polymeric acid, and allows it to ionise and donate protons, thereby behaving as a Bronsted-Lowry acid [35]. It is also the medium in which the setting reaction takes place and it is a component of the set cement. The last is an important but overlooked feature. All of the water incorporated initially as the solvent for the acidic polymer eventually becomes entrained in the set cement. There is no phase-separation on setting, and no expulsion of water as the cement hardens.

Maintaining the water balance early in the life of the cement is important. Many years ago it was shown that, if glass-ionomers are allowed to set in an atmosphere with a relative humidity of at least 80%, they showed no setting contraction and were dimensionally stable [36]. Loss of surface water can alter the appearance of glass-ionomer cements, leading to an unsightly chalky finish. This is due to the formation of microscopic cracks in the surface caused by local contraction as the water escapes [37]. To prevent this, clinicians are advised to cover the surface of newly placed glass-ionomers with some sort of barrier coating [37]. This may be either petroleum jelly or a varnish. The latter may consist either of a simple solution of film-forming resin in a solvent. On placement, the solvent evaporates readily, leaving the layer of resin behind as the barrier coating. Alternatively, a low-viscosity light-curable monomer may be used, and this is cured immediately on placement to create the impermeable barrier coating. The latter gives better results, because it forms a more continuous film and hence retains more water within the cement [37].

In the cement, the water undergoes some sort of interaction with the other chemical species present and becomes strongly bound. The distinction between strongly bound and loosely bound has been known about for some years since it was first advanced [38]. It is recognised as arbitrary, with loosely bound water being considered that which is lost by desiccation over a strong desiccant for 24 hours, or on heating at 105 °C for the same length of time, and strongly bound water being that which is not removed by these treatments. Despite these arbitrary definitions, these are useful distinctions as they show (a) that water occupies different locations within the cement and (b) that its distribution among these locations varies with time.

There seem to be a number of ways in which water becomes bound with glass-ionomer cements with time. One is by hydration of the cations released from the glass. Cations within the glass (Na^+^, Ca^2+^ or Sr^2+^, Al^3+^) are all present in the anhydrous state, yet are capable of co-ordinating strongly to water, and all will form strongly hydrated ions under appropriate conditions [39]. It seems likely that they do so within cements and that the hydrated ions formed are stable and able to retain their water molecules, even under desiccating conditions.

Studies have shown that, of the ions involved, aluminium binds most strongly to water molecules. In the solution state, it forms Al(H_2_O)_6_^3+^ ions, which have octahedral geometry [39]. This species is unlikely to form with glass-ionomer cements, but rather an octahedral structure involving carboxylate anions from the polymer and fluoride ions as well as some water is more probable.

Calcium and strontium ions are also important in the setting of glass-ionomers. Both have high co-ordination numbers in aqueous solution, either 7 or 8 [40, 41] depending on the method used to determine them. Like aluminium, such species are not anticipated to occur in glass-ionomer cements, but instead, calcium and strontium ions co-ordinated by varying numbers of carboxylate groups, fluoride ions and water molecules are expected. The fact that both ions are capable of such high co-ordination numbers with water suggests that plenty of potential binding sites become available as these ions pass from the glass to the matrix. Gradual occupation of at least some of the sites by water molecules could account for a significant amount of the bound water within set glass-ionomer cements.

Water is also able to bind to the ionised polyacid molecule [42]. Fundamental studies of polyelectrolytes, the class of polymer to which both polyacrylic acid and acrylic/maleic acid copolymer belong, have shown that a stable sheath of water molecules forms around the polyelectrolyte molecules in aqueous solution [42]. Such a sheath is likely to persist as the cement hardens and solidifies. Recent studies using neutron beams have confirmed that water is able to bind directly to the polyelectrolyte chain in glass-ionomer cements [43], confirming the earlier findings using less sophisticated experimental techniques.

Studies of the ratio of bound to unbound water in various dental cements have shown that all types contain an amount of bound water [38]. The fact that this is true of zinc polycarboxylate cements demonstrates that the proposed co-ordination of water to metal ions and the development of a hydration sheath around polyacrylate molecules are highly probable mechanisms of water-binding. It should be noted, however, that Zn^2+^ ions have only six co-ordination sites available [39], so this material will necessarily bind slightly less water than glass-ionomers. In fact, glass-ionomers bind considerable more water than zinc polycarboxylates, and this has led to the suggestion of additional binding mechanisms.

Some years ago, it was suggested that this was due to the extra hydration requirements of the silica gel formed on the surface of ion-depleted glass particles within the cement [44]. More recently, the possibility has been proposed that siloxane groups (-Si-O-Si-) in the surface of glass particles undergo reaction with water to form silanol groups (-Si-OH) [45]. This possibility is consistent with findings from FTIR spectroscopy, where reductions in the band due to siloxane at 1060 cm^−1^ have been observed as cements mature. There is also some evidence of bands due to silanol at 950 cm^−1^ and around 3740 cm^−1^ increasing, though this can be difficult to observe because of overlap with bands due to hydrogen-bonded water [46, 47].

Silanol groups have been observed on the surface of silica particles using FTIR [48] and their ability to form hydrogen bonds, both with each other [49] and with water [50] have been considered in molecular modelling studies. Results are complex, but broadly it has been shown that silanol groups are able to form both weak and strong hydrogen bonds with water, depending on their orientation with respect to the solid surface [50]. Where silanol groups are out-of-plane with the surface, they form strong hydrogen bonds to water molecules, which results in strongly adsorbed water on the surface, and the development of a pseudo-crystalline structure that has been described as “ice-like” [48]. If such species can form within glass-ionomer cements, they are likely to be stable to desiccation and to contribute to the overall population of bound water molecules.

Water itself is known to have a degree of mobility within cements and to be capable of diffusing through and out of glass-ionomers [51]. Diffusion coefficients indicate, not surprisingly, that such movement is slow, and this is one reason that maturation events involving water take 4–6 weeks to complete. Slow diffusion of water to relevant binding sites is likely to be the rate-determining step in the maturation process, as moving to equilibrium positions in the co-ordination sphere of metal ions or the hydration sheath of poly-anions is likely to be relatively rapid in both cases. Only hydration of siloxane groups via reaction, followed by formation of “ice-like” bound water layers seems likely to be comparably slow.

Overall, therefore, it seems that there is a wide variety of possible mechanisms of binding of water within glass-ionomer cements. All of them have been shown to be feasible experimentally, sometimes with similar materials rather than glass-ionomers themselves. Further work is necessary to confirm some of these proposed mechanisms, and also to determine the rate at which maturation reactions occur in modern glass-ionomer formulations.

## Ion-exchange bonding to the tooth surface

Glass-ionomer cements are naturally adhesive to teeth at all stages of their development. They owe their initial adhesion to the presence in them of polyacrylic acid or acrylic/maleic acid copolymer [3]. The hydrophilic nature of the cement paste causes it to fully wet the freshly prepared tooth surface. Adhesion develops rapidly after initial placement as hydrogen bonds are formed between the free carboxyl groups in the cement and strongly bound water layers on the tooth surface [51]. These hydrogen bonds are gradually replaced by ionic bonds involving cations such as calcium in the mineral phase of the surface of the tooth and carboxylate groups on the polymer [52].

After that, there is slow reaction between the surface layer of the tooth and the cement involving exchange of ions [53]. The result is a strong, chemically resistant interfacial layer that provides an adhesive interaction that is very durable and prevents leakage to the underlying natural tooth.

True chemical bonding between glass-ionomer cements and the tooth has been demonstrated experimentally on enamel and dentine using X-ray photoelectron spectroscopy [54]. This involves the mineral phase only, and does not include any measurable interaction with the collagen [55]. X-ray photoelectron spectroscopy is a high vacuum technique that does not allow clinically realistic conditions to prevail. However, more recently it has been confirmed that glass-ionomers undergo a chemical reaction with the mineral phase of teeth, using FTIR to study the reaction that occurs when ground tooth material was added to glass-ionomer dental cements [56]

The occurrence of ion-exchange to promote the formation of a chemically and mechanically strong interfacial layer was demonstrated some years ago [57]. This study employed teeth that had been filled with the commercial glass-ionomer Fuji IX (GC, Tokyo, Japan). The cement is formulated from a glass that contains no calcium and with strontium in its place.

**Figure 2.**
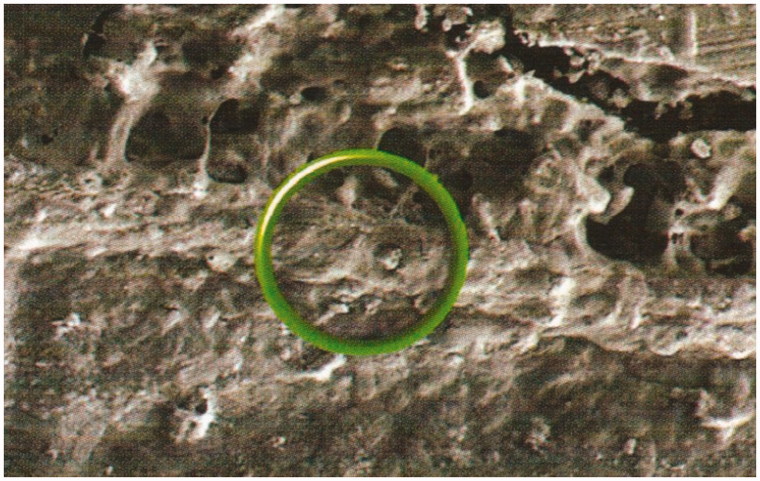
Ion-exchange interfacial layer formed between glass-ionomer cement (Fuji IX) and a natural tooth over five years.

After five years, teeth filled with this cement were extracted for orthodontic reasons, and the teeth examined by scanning electron microscopy (Figure 2). The resulting interfacial zone is clearly visible. Elemental analysis showed that it contained both calcium and strontium, a finding possible only because of diffusion of calcium from the tooth mineral and strontium from the cement into the interfacial zone. The structure formed by this process causes the cement to adhere strongly to the tooth.

The development of the ion-exchange layer relies on the respective cations being sufficiently mobile to diffuse into the interfacial zone and there react with appropriate anions to form an insoluble structure. Glass-ionomer cements have been shown to be able to release various ions under neutral conditions for some weeks after the initial set is complete, though release of calcium was very low under neutral conditions [58]. Fluoride release is also known to be able to continue for at least five years [59], and to be released by a mechanism that includes long-term sustained diffusion [60]. Hence there is evidence that, although the properties of glass-ionomer cements change as they mature, ions are able to diffuse slowly through them. This may affect not only the interfacial layer, as observed, but also the size of the apparent pores within the cement and may be the mechanism by which such pores become smaller with time [27].

## Conclusions

This review has shown that there are various maturation processes in glass-ionomer cements. They take place over the first month to 6 weeks of a cement’s existence and they generally combine to improve the physical properties of the cement. Materials become stronger, less susceptible to water loss and surface crazing, and their appearance improves as translucency increases. These processes occur relatively slowly and appear to be controlled by the rate at which ions and water are able to diffuse through the cement. Movement of water has been demonstrated experimentally in water-loss experiments carried out under severely desiccating conditions, and movement of ions has been shown by the formation of the ion-exchange bonding layer at the interface with the tooth; it has also been shown in fluoride-release studies.

Current views on the underlying mechanisms of the various maturation changes have been described. Much of the work on these effects was carried out several years ago using some of the earliest versions of glass-ionomer cements. There is a need for these studies to be updated and for information on contemporary materials to be obtained. It is especially important to establish the extent to which these maturation changes occur in modern cements, and also to determine how rapidly they take place.
